# Calcaneal Ultrasound Attenuation: Does the Region of Interest and Loading Influence the Repeatability of Measurement?

**DOI:** 10.1007/s00223-025-01357-x

**Published:** 2025-03-10

**Authors:** Aaron P. Robertson, Brendan J. Jones, Christian M. Langton, Scott C. Wearing

**Affiliations:** 1 School of Biomedical Sciences, Queensland University of Technology, Brisbane, 4000 Australia; 2 Brisbane Private Imaging, Brisbane, Australia; 3 School of Clinical Sciences, Queensland University of Technology, Brisbane, Australia; 4 Griffith Centre of Biomedical and Rehabilitation Engineering, Griffith University, Gold Coast, Australia; 5 School of Medicine and Health Sciences, Technical University of Munich, Bavaria, Germany

**Keywords:** Reproducibility of results, Quantitative ultrasound, Bone density, Bone quality

## Abstract

Current calcaneal quantitative ultrasound systems assess different regions of interest (ROI), under different levels of lower limb loading, yield different parameter values, and are likely prone to different levels of error. This study evaluated the repeatability of measures of frequency-dependent attenuation (FDA, 0.3–0.8 MHz) at three calcaneal ROI, Brooke–Wavell (BW), Jaworski (JA), and foot gauge (FG), under four loading conditions (non-weightbearing, semi-weightbearing, bipedal stance, and unipedal stance). FDA in the calcaneus was assessed in 20 healthy participants (mean (± SD) age, 41.7 ± 19.6 years; height, 1.70 ± 0.16 m; and weight, 70.1 ± 23.0 kg) using a custom-built transmission-mode ultrasound system. Reliability was evaluated using the standard error of measurement (SEM) and limits of agreement (LA) and tolerance (95%TL). Differences in mean FDA values between ROI, loading, and measurement occasions were assessed using a repeated measures ANOVA (α = .05). Mean FDA values ranged between 58.0 ± 32.0 and 77.2 ± 27.6 dB/MHz across all conditions. Repeatability of FDA was dependent on the ROI examined and tended to improve with weightbearing. The narrowest limits for 95%TL ranged between ± 15.1 dB/MHz (JA SWB) and ± 62.7 dB/MHz (BW NWB) across sites. The SEM was approximately 10 dB/MHz for both FG and JA during non-weightbearing and was reduced to around 5 dB/MHz with full weightbearing. This study demonstrates that, although measures of ultrasound FDA are dependent on the ROI, lower limb loading may be a useful method to improve the repeatability of FDA measurements.

## Introduction

Osteoporosis is a metabolic bone disorder characterised by low bone mass and abnormal bone architecture resulting in an increased susceptibility to bone fracture, as evidenced by the large body of knowledge in adult populations [1]. There is increasing recognition that bone disorders, such as osteoporosis, also affect children: not only as a primary aetiology, but also secondary to chronic illness, musculoskeletal injury, medication, and lifestyle issues [2]. While dual-energy X-ray absorptiometry (DXA) is the most widely used technique for evaluating osteoporosis and fracture risk in adults [3], its use to quantify bone properties in children and adolescence has several limitations. In particular, DXA-based estimates of bone mineral density are highly dependent on bone morphology, body size, and skeletal maturity [4]. In addition, normative data in children and adolescents are sparse, presumably reflecting the use of ionising radiation, high cost, and low accessibility associated with use of DXA [2]. Such shortcomings have subsequently fuelled research into alternative methods for quantifying bone status across all age groups.

Quantitative ultrasound (QUS) is a useful alternative to DXA for quantifying bone status. In contrast to DXA, it is relatively low cost, radiation free, and portable [5]. Unlike medical ultrasonography, which provides an image, QUS assesses the interaction between ultrasound waves and bone tissue to provide quantifiable variables associated with the structure, elastic modulus, and density of the bone [3]. The term QUS describes several anatomical measurement sites, primarily the calcaneus, and different measurement parameters, primarily ultrasound velocity (m/s), and frequency-dependent attenuation (FDA, dB/MHz). FDA represents the linear increase in ultrasound attenuation over a defined frequency range; In general, this has been 200 kHz to 600 kHz and referred to as broadband ultrasonic attenuation (BUA). A limitation of QUS is the lack of standardisation of measurement. Currently, QUS devices assess different calcaneal regions of interest (ROI), yield different parameter values, and are likely prone to different levels of error [6]. The variation in skeletal site and error hampers meaningful attempts to compare bone measurements across groups reported in the literature. Calcaneal ROI for measurement with QUS have typically targeted the posterior aspect of the bone, where cortical surfaces are considered to be generally flat and parallel [7].

Current calcaneal QUS devices generally provide measurements at a fixed distance from the posterior and inferior aspects of the heel [8, 9]. Hence, ROI differ between people depending on their foot size, which may result in the interrogation of sites of varied calcaneal cortical thickness, bone volume fraction, trabecular orientation, and level of ossification [8, 10–14]. For instance, Brooke-Wavell et al. [15], showed that small variations in ROI resulted in the evaluation of different trabecular structures and contributed to measurement error. Similarly, differences in the thickness of overlaying soft tissue (such as the volume of fatty tissue or level of oedema) has have been shown to influence attenuation and introduce error [8, 16]. While the vast majority of QUS devices use a fixed global site for measurement of QUS parameters, some studies have provided anatomically located ROI to reduce measurement-related issues associated with between-subject variation in skeletal site measurement [5, 10, 16].

Previous studies also generally perform QUS measurements without consideration of the physical loading environment of the calcaneus. Most studies have used QUS systems that require the calcaneus to be partially loaded without the consideration for individual differences in the weight of each lower limb. Implicit to these studies, therefore, is the assumption that physical loading does not influence the ROI location nor the propagation of ultrasound through the calcaneus. However, in linear isotropic materials, the stress state of a medium is known to impact the phase velocity and attenuation of ultrasonic waves [17], a phenomenon which has also been proposed to occur in bone [18]. It is possible, therefore that varied lower limb load may also affect the reproducibility of measures of calcaneal FDA. Although the precision, or closeness of agreement between independent QUS measures, under specific conditions, is well documented [5, 15, 19–21], their reproducibility or precision in different laboratories with different operators, using different equipments, has been questioned [22]. To the best of the author’s knowledge, no study has compared the effect of loading on variability of calcaneal FDA at different ROI of the calcaneus in vivo.

The aim of this study, therefore, was to evaluate the repeatability of measures of FDA at three ROI of the calcaneus and under varying loading conditions in a group of healthy children, adolescents, and adults. Given the broad range of precision values obtained for FDA within the literature [22], this study specifically evaluated absolute, rather than relative, measures of reliability. Moreover, to evaluate potential artefacts arising from skin movement at each ROI, the central location of each ROI was determined relative to the underlying osseous anatomy under extreme loading conditions via the use standard foot radiographs. It was hypothesised that FDA measurement values would be site dependent, and repeatability would improve with increased loading. Consistent with previous research investigating the difference between calcaneal FDA between healthy populations and their controls [5, 23–28], the authors considered 15 dB/MHz to be the minimal detectable difference that is clinically important. The effect of potential confounding factors on measurement values of FDA, including load-induced movement of soft tissues relative to the underlying bone, was also explored.

## Materials and Methods

A repeated-measures study design was used to assess the reliability of FDA measured on two occasions at three different ROI and with varied levels of lower limb loading.

### Participants

A convenience sample of children, adolescents, and adults (*n* = 20) were recruited from the greater Brisbane metropolitan area. As recommended for method-comparison studies [29], a broad age range was evaluated to ensure that FDA measurements encompassed the full physiological range of values reported within the literature [5, 30]. Participants ranged in age from 8 to 71 years, with a median age of 42.0 years. Four of the 20 participants were aged less than 18 years, while four were greater than 60 years. The mean (± SD) height and weight of participants were 1.70 ± 0.16 m and 70.2 ± 23.0 kg, respectively. Of the twenty participants recruited for this study, thirteen were male and seven were female. No participant reported a medical history of autoimmune disease, bone disease, cancer, calcaneal fracture, or foot surgery. Sample size for the study was estimated based on previously published data for healthy children and adults [31]. A sample size of 20 participants was found to be sufficient to achieve a point estimation of 0.30 in the width of the 95% confidence interval, assuming a correlation of 0.80 among repeated measures [32]. Moreover, participant numbers also provided sufficient statistical power (*β* = 0.20) to detect a 15 dB MHz difference in measures of FDA, at an α-level of 0.05. Hence, the study was statistically powered to not only determine stable absolute estimates of reliability but to also identify differences equivalent to the minimum important difference reported within the literature for FDA in children and adult populations [23–28]. Consent was obtained from participants following a verbal and written explanation of the methods. Assent was obtained from participants under the age of 18 years, along with consent from the legal guardian. Ethical approval for all aspects of the project was received following review by the University Human Research Ethics Committee (1500001041).

### Equipment and Measurement

FDA (dB/MHz) at the calcaneus was determined using a gel coupled ‘dry’ contact transmission-mode ultrasound system. The system included a portable signal generator and receiver (Omniscan MX, Olympus Australia Pty Ltd, Notting Hill VIC) and two coaxially aligned 1 MHz transducers (0.5 MHz bandwidth), each 1.27 cm in diameter (Olympus NDT, MA, USA). The transducers incorporated nominally 10 mm silicone coupling pads and were mounted in an adjustable calliper. Transducer separation, from pad to pad, was measured using a digital Vernier gauge with a resolution of 10 µm. The Omniscan MX system generated a high-voltage pulse (90 V), and received signals were digitised at 100 MHz (10 bits). Radio-frequency signals were subsequently sampled at 20 MHz providing 640 data points over a measurement range of 50 mm at a system velocity of 1480 m/s. FDA was calculated, relative to degassed water (at room temperature), over a 0.3–0.8 MHz frequency range using established methods [33] and custom software (Matlab software; MathWorks Inc., Natick, Massachusetts, USA).

### ROI

**Fig. 1:**
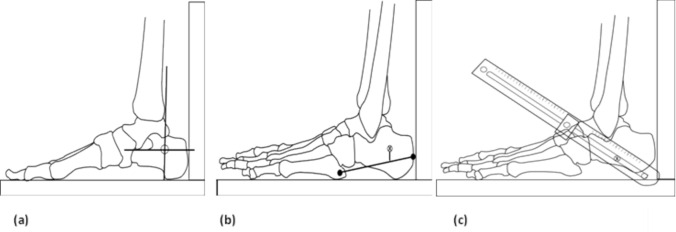
Illustration of the three ROI used for FDA assessment: **a** Brooke-Wavell (BW) [15], **b** Jaworski (JA) [5], and **c** foot gauge (FG)

Three different ROI were evaluated (Fig. 1). Two anatomical ROI (BW and JA) have been previously detailed elsewhere [5, 15] and require knowledge and palpation of relevant anatomy. A third, novel ROI (FG) was also tested. A custom-built foot gauge was used to identify a ROI that was one third of the distance between the dorsal–anterior ankle and the most infero-posterior aspect of the heel. The foot gauge was designed to allow for identification of a standardised anatomical location while considering differences in foot shape and size.

### Protocol

Participants reported to the laboratory wearing lightweight, comfortable clothing. For each ROI, ultrasound data were collected under four different loading conditions: (1) non-weightbearing (NWB), (2) semi-weightbearing (SWB), in which the participant was seated with their knee and ankle positioned perpendicular to the supporting surface, (3) weight-bearing during dual limb stance (DLS), and (4) weight-bearing during single limb stance (SLS). Prior to ultrasound measurement, the skin was prepared with conventional ultrasound gel. Calcaneal ultrasound signals were subsequently recorded at each ROI under each loading condition. For each participant, replicate measures at each ROI were made with incremental loading, from NWB through to SLS, with each measurement taking no longer than 30 s to complete.

To evaluate the location of each ROI relative to the underlying osseous anatomy, spherical radiopaque markers (Ø, 1.5 mm) were fixed to the skin at the centroid of each ROI. Standard anteroposterior and lateral foot radiographs were then acquired (MULTIX Impact C1, Siemens, Brisbane, Australia) using a radiation exposure equivalent to 2.5 mA s^−1^, an intensity of 52 kVp, and a focal distance of 1 m. To minimise the exposure of participants to ionising radiation, radiographic images were acquired only under the most extreme weightbearing conditions; namely, NWB and SLS. Anteroposterior and lateral images were acquired with the central ray focused at 50% of foot length. Spatial distortion within the imaging system was minimised using a rectilinear calibration grid (32 × 32 cm) positioned within the object plane and perpendicular to the central ray [34], in combination with a distortion correction procedure [35]. The root-mean-square error for repeated linear measures of nine known calibration structures positioned within the field of view was less than 0.2 mm using this method. Radiographic images were evaluated using custom software (Matlab software; MathWorks Inc., Natick, Massachusetts, USA). The anterior, posterior, inferior, and superior aspects of the calcaneus were manually digitised and fit with a rectangular bounding box. The coordinates of the centroid of each ROI were expressed relative to the posterior and inferior origin of the bounding box and normalised to calcaneal length and height.

### Statistical Analysis

The SPSS™ statistical package (SPSS, Chicago, IL) was used for all statistical procedures. Shapiro-Wilke tests were used to evaluate underlying assumptions of normality. Outcome variables were determined to be normally distributed, and hence, means and standard deviations have been used as summary statistics. Absolute reliability was estimated using the Standard Error of Measurement (SEM), and 95% Limits of Agreement (LA) as outlined by Bland and Altman [36]. Plots were visually and statistically evaluated for evidence of heteroscedasticity using the approach outlined by Darlington [37]. Fixed bias between methods was assessed using one-sample t-tests, while proportional bias was evaluated using ordinary least squares regression. In the absence of proportional bias and heteroscedasticity, 95% limits of agreement were calculated using the method outlined by Ludbrook [38], which includes adjustments for small sample sizes (*n* < 60). In addition, 95% tolerance limits (95%TL), which represent the range within which a single, new, observation taken from the same population would be expected to lie, were calculated using exact parametric confidence intervals as outlined by Carkeet [39]. The 95%TL are conceptually the same as the *Minimum Detectable Change* (MDC_95%_), representing the minimum change that is likely to reflect the true change, at and individual level (with 95% confidence), instead of measurement error alone [40].

Potential differences in FDA between each ROI and loading condition were assessed using a two-way repeated measures analysis of variance (ANOVA) within a generalised linear modelling framework. In each case, ROI (BW, JA, FG) and loading (NWB, SWB, DLS and SLS) were treated as within-subject factors. The effect of weightbearing on the location of the centroid of each ROI was investigated using a one-way repeated measures ANOVA. Mauchly’s test of sphericity was used to assess assumptions of sphericity of the variance–covariance matrix. Where significant departures from sphericity occurred, the most conservative adjustment with Greenhouse–Geisser Epsilon was used [41]. Statistically significant main effects were evaluated using simple contrasts. An alpha level of 0.05 was used for all tests of significance.

## Results

The SEM for each ROI and under each loading condition are demonstrated in Table 1. The SEM of FDA measured at FG and JA ROIs were approximately 8.4 dB/MHz during NWB and tended to reduce to approximately 5.3 dB/MHz during DLS.

**Table 1:** Standard error of measurement (SEM, dB/MHz) for each region of interest and under each loading condition

	BW		FG		JA	
	SEM	SEM%	SEM	SEM%	SEM	SEM%
NWB	12.6	16	7.9	14	8.8	12
SWB	9.3	13	7.8	12	3.6	5
DLS	9.1	12	4.5	8	4.2	7
SLS	15.1	21	5.1	10	5.4	9

*BW* Brooke-Wavell [15]; *JA* Jaworski [5]; *FG* foot gauge; *NWB* non-weightbearing; *SWB* semi-weightbearing; *DLS* dual limb stance; *SLS* single limb stance

There was no evidence of fixed or proportional bias for any ROI under any loading condition (Table 2). However, the difference between replicate measures (fixed bias) at JA during SLS condition approached statistical significance (t_19_ = 2.1, *P* = 0.054). As demonstrated in Table 2, the narrowest 95%TL for repeated measures of FDA ranged between ± 15.1 dB/MHz and ± 62.7 dB/MHz across all sites. The FG and JA typically demonstrated the narrowest 95%TL across different loading conditions. Weightbearing tended to improve the 95%TL for repeated measures at FG and JA, decreasing from ± 36.6 dB/MHz for JA during NWB to ± 21.4 dB/MHz during SLS.

**Table 2:** Fixed bias, proportional bias, 95% limits of agreement (95%LA), and 95% tolerance limits with 95% confidence (95%TL) for ROI and loading condition

		Fixed bias			Proportional bias			± 95%LA	± 95%TL
	Bias	95%CI_FB_	t_19_	P_FB_	(r)_PB_	95%CI_PB_	P_PB_		
NWB–BW	−0.4	(−8.8 to 7.9)	−0.11	.92	−0.04	(−0.48 to 0.41)	.85	35.0	52.5
NWB–FG	1.2	(−4.1 to 6.4)	0.47	.64	−0.15	(−0.56 to 0.31)	.52	21.9	32.9
NWB–JA	4.0	(−1.8 to 9.8)	1.44	.17	0.19	(−0.27 to 0.59)	.42	24.4	36.6
SWB–BW	3.6	(−2.5 to 9.8)	1.23	.23	0.21	(−0.26 to 0.60)	.38	25.7	38.7
SWB–FG	−0.7	(−5.9 to 4.5)	−0.28	.78	−0.03	(−0.47 to 0.42)	.90	21.7	32.6
SWB**–**JA	−0.9	(−3.3 to 1.4)	−0.83	.42	0.36	(−0.10 to 0.69)	.12	10.0	15.1
DLS–BW	1.5	(−4.6 to 7.6)	0.51	.61	0.08	(−0.37 to 0.51)	.73	25.4	38.1
DLS–FG	−0.8	(−4.9 to 3.3)	−0.42	.68	−0.06	(−0.49 to 0.39)	.79	17.1	25.7
DLS–JA	1.2	(−1.6 to 3.9)	0.89	.39	−0.25	(−0.63 to 0.21)	.28	11.6	25.6
SLS–BW	6.9	(−3.1 to 16.9)	1.44	.16	−0.06	(−0.49 to 0.39)	.80	41.8	62.7
SLS–FG	2.9	(−0.1 to 5.9)	2.06	.05	0.15	(−0.32 to 0.56)	.53	12.5	18.7
SLS–JA	3.0	(−0.4 to 6.4)	−6.4	.08	0.19	(−0.28 to 0.58)	.44	14.3	21.4

*NWB* non-weightbearing; *SWB* semi-weightbearing; *DLS* dual limb stance; *SLS* single limb stance; *BW* Brooke-Wavell [15]; *JA* Jaworski [5]; *FG* foot gauge

**Fig. 2:**
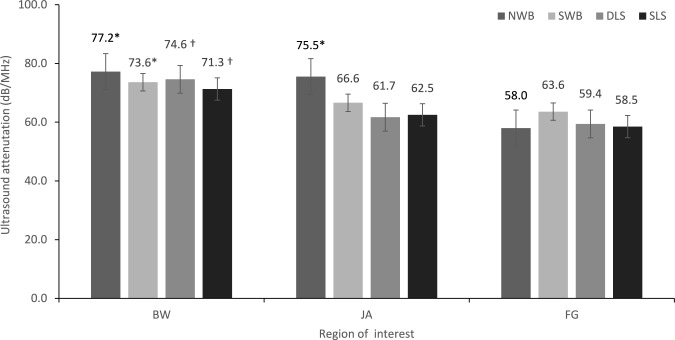
Mean (SD) FDA for the Brooke–Wavell (BW) [15], Jaworski (JA) [5], and foot gauge (FG) ROI measured during non-weightbearing (NWB), semi-weightbearing (SWB), dual limb stance (DLS), single limb stance (SLS). † indicates significantly different from all other ROI for the same loading condition (*P* <  .05). * indicates significantly different from FG for the same loading condition (*P* <  .05)

The mean and standard deviation for FDA at each ROI under each loading condition are presented in Fig. 2. There was a significant main effect for ROI (F_1.73, 32.92_ = 11.8, *P* =  < 0.001, partial η^2^ = 0.38). Simple contrasts revealed that the BW ROI was significantly different compared to the FG ROI under NWB (F_1,19_ = 13.5, *P* = 0.002, partial η^2^ = 0.42), SWB (F_1,19_ = 4.8, *P* = 0.04, partial η^2^ = 0.20), DLS (F_1,19_ = 11.85, *P* = 0.003, partial η^2^ = 0.39), and SLS (F_1,19_ = 8.66, *P* = 0.008, partial η^2^ = 0.31) and at the JA ROI under DLS (F_1,19_ = 7.24, *P* = 0.014, partial η^2^ = 0.28) and SLS (F_1,19_ = 7.20, *P* = 0.015, partial η^2^ = 0.28). In addition, the JA ROI significantly differed to the FG ROI under NWB (F_1,19_ = 11.94, *P* = 0.003, partial η^2^ = 0.39). There was no statistically significant main effect for load nor significant interaction between load and site, despite approaching statistical significance (*P* = 0.059).

**Fig. 3:**
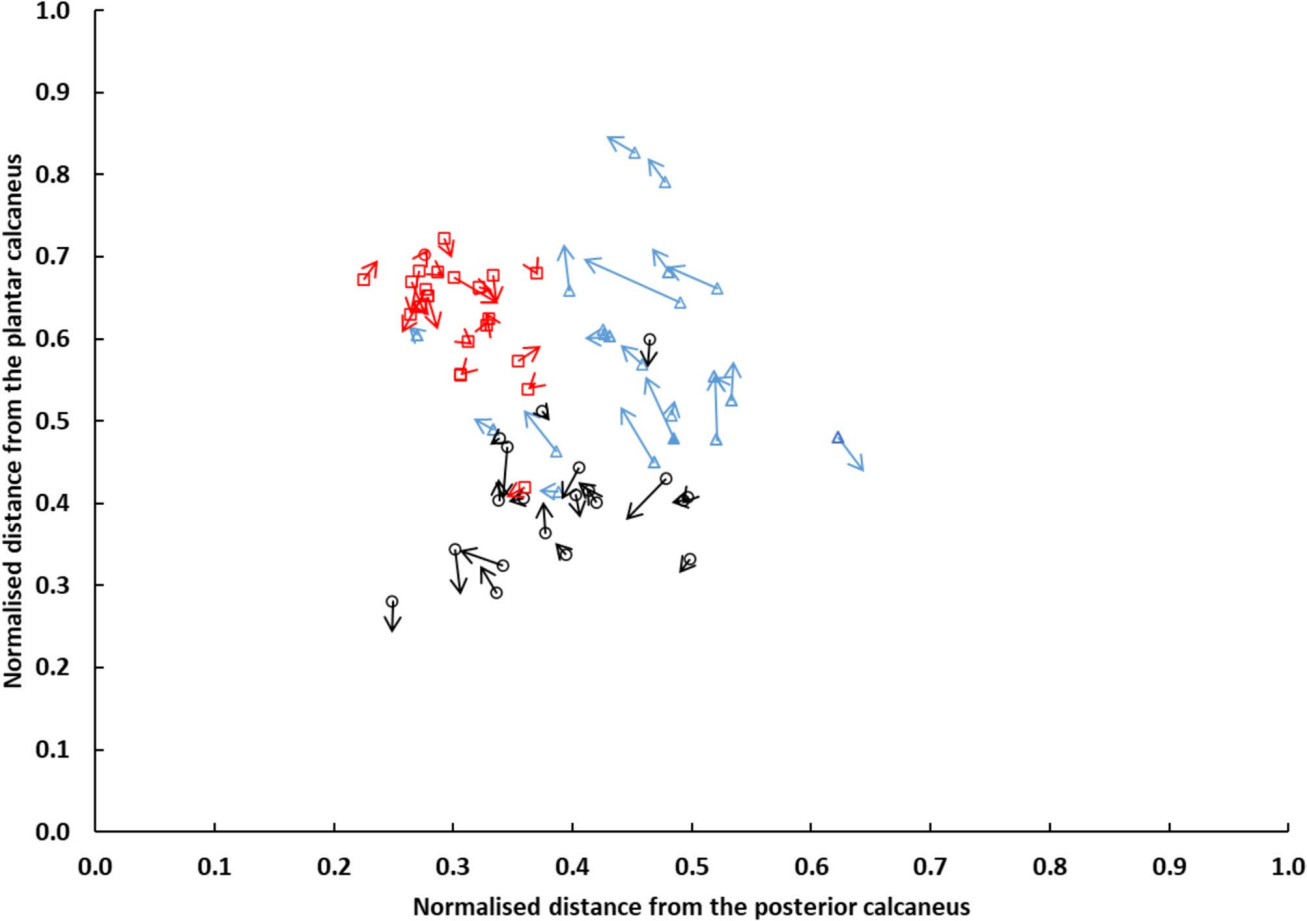
The position of the spherical radiopaque marker at the centroid for the Brooke–Wavell (blue triangle), Jaworski (red square), and foot-gauge (black circle) ROI relative to the posterior and inferior surface of the calcaneus during non-weightbearing for each participant. Arrows indicate the resultant displacement of the centroid during single limb stance. The size of the arrow reflects the magnitude of the displacement, while the arrowhead represents the change in direction of the centroid with weightbearing

The mean and standard deviation for the coordinates of each ROI during NWB and SLS are shown in Table 3. There was a significant difference in mean displacement of the centroid of each ROI with weightbearing (F_2,38_ = 11.7, *P* =  < 0.001, partial η^2^ = 0.38). Simple contrasts revealed that displacement at the BW ROI (2.6 ± 2.4 mm) was significantly greater than at the FG ROI (1.6 ± 1.5 mm, F_1,19_ = 10.2, *P* = 0.005, partial η^2^ = 0.35) and JA ROI (1.4 ± 1.0 mm, F_1,19_ = 26.2, *P* =  < 0.001, partial η^2^ = 0.58). However, there was no significant difference in displacement between FG and JA ROIs. The dispersion of the centroids within FG and BW ROI was greater than that of the JA ROI. At the level of the individual, the direction and magnitude of displacement were not consistent within or between ROI with weightbearing (Fig. 3).

**Table 3:** Mean (SD) coordinates for each ROI, Brooke–Wavell (BW) [15], Jaworski (JA) [5], and foot gauge (FG), under each loading condition NWB (non-weightbearing), SLS (single limb stance)

Region of interest	BW		FG		JA	
Loading condition	NWB	SLS	NWB	SLS	NWB	SLS
Distance from posterior calcaneus (mm)	27.2 (21.7)	26.4 (21.0)	29.8 (7.3)	29.5 (8.8)	23.3 (4.6)	23.8 (6.2)
Distance from plantar calcaneus (mm)	33.4 (8.1)	34.8 (9.8)	23.1 (5.4)	22.4 (6.6)	36.2 (4.4)	35.1 (8.2)

## Discussion

This study evaluated the repeatability of measures of FDA at three ROI of the calcaneus and under varying loading conditions using a contact transmission-mode ultrasound system in a group of healthy children, adolescents, and adults. Overall, the 95%TL for FDA differed between ROI, with the narrowest 95%TL (± 15.1 dB/MHz and ± 18.7 dB/MHz) observed at the JA ROI during SWB and FG ROI during SLS, respectively. Although measures of FDA were ROI dependent, lower limb loading tended to lower FDA and improve the repeatability of measurements across two of the three ROI evaluated. For instance, the 95%TL for repeated measures at the JA ROI were 1.7 times narrower during SLS than NWB. Hence, calcaneal loading may be a useful method to improve the minimum detectable difference of the measurement. However, why loading tended to improve repeatability at two of the three ROIs is not clear. One possible explanation is that the movement of the calcaneus becomes more constrained with increasing weightbearing. This concept is supported within the literature where Ito et al. [42] demonstrated that calcaneal movement is more restrained with increasing load. As to why repeatability did not improve at the BW ROI, this site anatomically overlays the tarsal tunnel, which is composed of several extrinsic flexor tendons, hence, with weightbearing, there is potentially increased relative skin movement. However, further research is needed to better understand this relationship.

Despite ex vivo studies demonstrating the deformation of the microstructure of trabecular bone under physical loading [43], to our knowledge only one, in vivo, cross-sectional study has investigated the effect of physical loading on the calcaneus with QUS [18]. Liu et al. [18] reported that self-weight loading (i.e. standing) resulted in a small reduction in calcaneal FDA values (within the frequency range designated by their proprietary clinical scanner) in a group of pre- and post-menopausal women. However, we observed no statistically significant effect of load on FDA measures across the three ROI (*P* = 0.06, partial η^2^ = 0.11). Although comparisons between studies are hampered by the lack of standardisation of measurements, it should be noted that the anatomical location of each ROI evaluated in the current study tended to change with respect to the calcaneus during weightbearing (Fig. 3). The largest displacement occurred at the BW ROI (2.6 ± 2.4 mm), which was approximately one and a half times that of the other two ROI. Moreover, although the BW ROI tended to move superiorly and posteriorly with weightbearing for the majority of participants, the direction of displacement with weightbearing was neither consistent within nor between ROI. Hence, when comparing across weightbearing conditions, specifically between NWB and SLS, it is important to recognise that the same site is not being measured. Although our findings tend not to support those of Liu et al. [18], it is noteworthy that in the current study, loading did tend to improve repeatability of measures of FDA at the FG and JA ROI.

As demonstrated in Fig. 3, the scatter of individual-level data around the centroid of each ROI provides insight into the consistency of the operator in identifying the anatomical location of each ROI between participants. Measurement sites were more closely clustered together for JA ROI compared to the BW or FG ROI, indicating that the JA ROI was more consistently identified. Unlike the BW and JA ROI, which require clinical palpation and detailed knowledge of the underlying anatomy of the foot, a novel foot gauge was also evaluated in this study in an attempt to standardise the anatomical location of the ROI by accounting for potential differences in foot shape and size. The foot gauge was designed to define a ROI that was one third of the distance between the dorsal point of the anterior ankle and the most inferior and posterior aspect of the plantar surface of the heel. However, the spread of the data points, as denoted by the standard deviation of the coordinates for each ROI were comparatively larger than that for the JA ROI. Despite this, the foot gauge did reduce the variability of the spread of the coordinates compared to the BW ROI and did appear to identify a location that had comparatively little soft-tissue movement and minimal differences in FDA values between NWB and SLS.

This study had several limitations which should be considered when interpreting the results. First, we evaluated a healthy cohort of children and adults to ensure a broad range of FDA values. Hence, further research is required to confirm whether the 95%TL of FDA with repeated measurement is comparable in a cohort presenting with bone pathology. It is anticipated that cohorts with certain bone pathologies would have lower FDA values than controls, with previous research demonstrating that calcaneal FDA in osteoporotic children significantly differed from their matched controls [5]. However, given the non-linear dependance of FDA on bone porosity and the associated absorption and scattering phenomena, as referred to by Njeh et al. [44], the absolute reliability of FDA in cohorts presenting with bone disorders may be subjected to increased levels of error. Second, we assessed intra-rater and intra-day reliability and, as such, cannot comment on the reproducibility of measures of FDA in different laboratories with different operators.

## Conclusion

This study confirmed that measures of calcaneal FDA are ROI-dependent and identified, for the first time, that calcaneal loading may be a useful method to improve the repeatability of FDA measurements in vivo. FDA measured at the BW ROI differed significantly from that measured at the FG and JA ROIs and was the least repeatable estimate. Even under the most repeatable loading condition (DLS), the 95%TL for repeated measures of FDA at BW (± 38 dB/MHz) was more than twice the clinically meaningful difference reported within the literature (≈15 dB/MHz). The reliability of the JA and newly developed FG ROI, in contrast, approached the clinically meaningful differences in FDA, particularly under SBW and SLS loading conditions. These findings have direct implications for calcaneal FDA protocols used to quantify bone status.
